# Rapid Trio Exome Sequencing for Autosomal Recessive Renal Tubular Dysgenesis in Recurrent Oligohydramnios

**DOI:** 10.3389/fgene.2021.606970

**Published:** 2021-06-21

**Authors:** Shin-Yu Lin, Gwo-Tsann Chuang, Chien-Hui Hung, Wei-Chou Lin, Yung-Ming Jeng, Ting-An Yen, Karine Chang, Yin-Hsiu Chien, Wuh-Liang Hwu, Chien-Nan Lee, I-Jung Tsai, Ni-Chung Lee

**Affiliations:** ^1^Department of Obstetrics and Gynecology, National Taiwan University Hospital, Taipei, Taiwan; ^2^Department of Pediatrics, National Taiwan University Hospital, Taipei, Taiwan; ^3^Department of Pathology, National Taiwan University Hospital, Taipei, Taiwan; ^4^Department of Medical Genetics, National Taiwan University Hospital, Taipei, Taiwan

**Keywords:** oligohydramnios, *AGT* gene, autosomal recessive renal tubular dysgenesis, angiotensinogen, whole exome sequencing (WES)

## Abstract

Oligohydramnios is not a rare prenatal finding. However, recurrent oligohydramnios is uncommon, and genetic etiology should be taken into consideration. We present two families with recurrent fetal oligohydramnios that did not respond to amnioinfusion. Rapid trio-whole-exome sequencing (WES) revealed mutations in the *AGT* gene in both families within 1 week. The first family had a compound heterozygous mutation with c.856 + 1G > T and c.857-619_1269 + 243delinsTTGCCTTGC changes. The second family had homozygous c.857-619_1269 + 243delinsTTGCCTTGC mutations. *AGT* gene mutation may lead to autosomal recessive renal tubular dysgenesis, a rare and lethal disorder that can result in early neonatal death. Both the alleles identified are known alleles associated with pathogenicity. Our findings suggest that trio-WES analysis may help rapidly identify causative etiologies that can inform prompt counseling and decision-making prenatally.

## Introduction

Oligohydramnios is defined as an amniotic fluid index (AFI) of less than 5 cm or a single deepest pocket of amniotic fluid below 2 cm (Manning et al., 1980). The fetal prognosis in oligohydramnios varies depending on several factors, including the underlying causes, the severity, and the gestational age at which oligohydramnios occurs (Rabie et al., 2017). Because an adequate volume of amniotic fluid is critical to normal fetal movement and lung development and for protecting the fetus and umbilical cord from uterine compression, pregnancies complicated by oligohydramnios with any cause are at risk for fetal deformation, pulmonary hypoplasia, and umbilical cord compression (Shipp et al., 1996).

Oligohydramnios may be idiopathic or have a maternal, fetal, or placental cause (Hesson and Langen, 2018). By 18 weeks of gestation, fetal urine becomes a major component of amniotic fluid, and the fetus begins to swallow amniotic fluid (van Otterlo et al., 1977; Abramovich et al., 1979). Therefore, fetal renal/urinary system disorders are important causes of oligohydramnios in the second trimester, including intrinsic renal disorders (e.g., bilateral renal agenesis or renal tubular dysgenesis) and obstructive lesions of the lower urinary tract (e.g., posterior urethral valves and urethral atresia) (Moxey-Mims and Raju, 2018). However, recurrent second-trimester oligohydramnios is a rare condition, and genetic etiology should be taken into consideration (Shipp et al., 1996; Supplementary Table 1).

In this study, we report recurrent oligohydramnios in two families with two pregnancies in the second trimester complicated by neonatal death. To investigate the genetic cause of recurrent oligohydramnios with non-consanguineous parents, sequential detection including whole-exome sequencing (WES) was performed to obtain a clear diagnosis.

## Case Description

### Family 1

**FIGURE 1:**
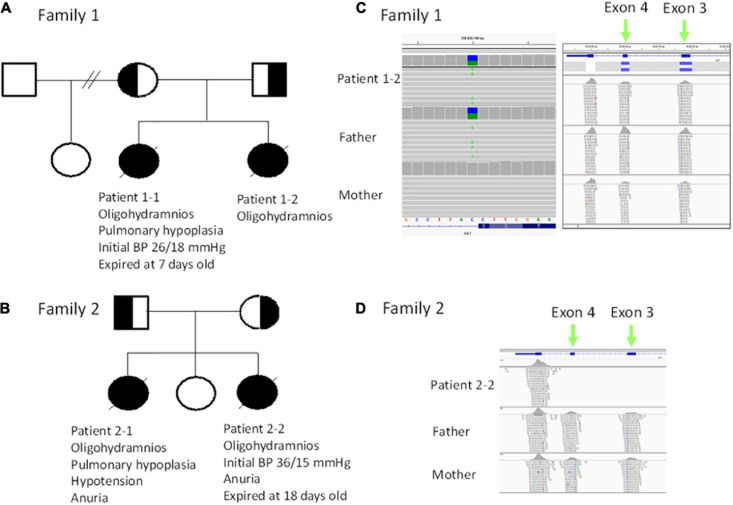
**(A)** Pedigree of family 1. **(B)** Pedigree of family 2. **(C)** Trio exome sequencing revealed a heterozygous c.856 + 1G > T mutation (in the reference is C, alteration is A) patient 1–2 and the father (left panel), while a heterozygous deletion (blue bar) in exon 3 and exon 4 was noted in patient 1–2 and mother. **(D)** Trio exome sequencing revealed a homozygous deletion in exon 3 and exon 4 in patient 2–2.

**FIGURE 2:**
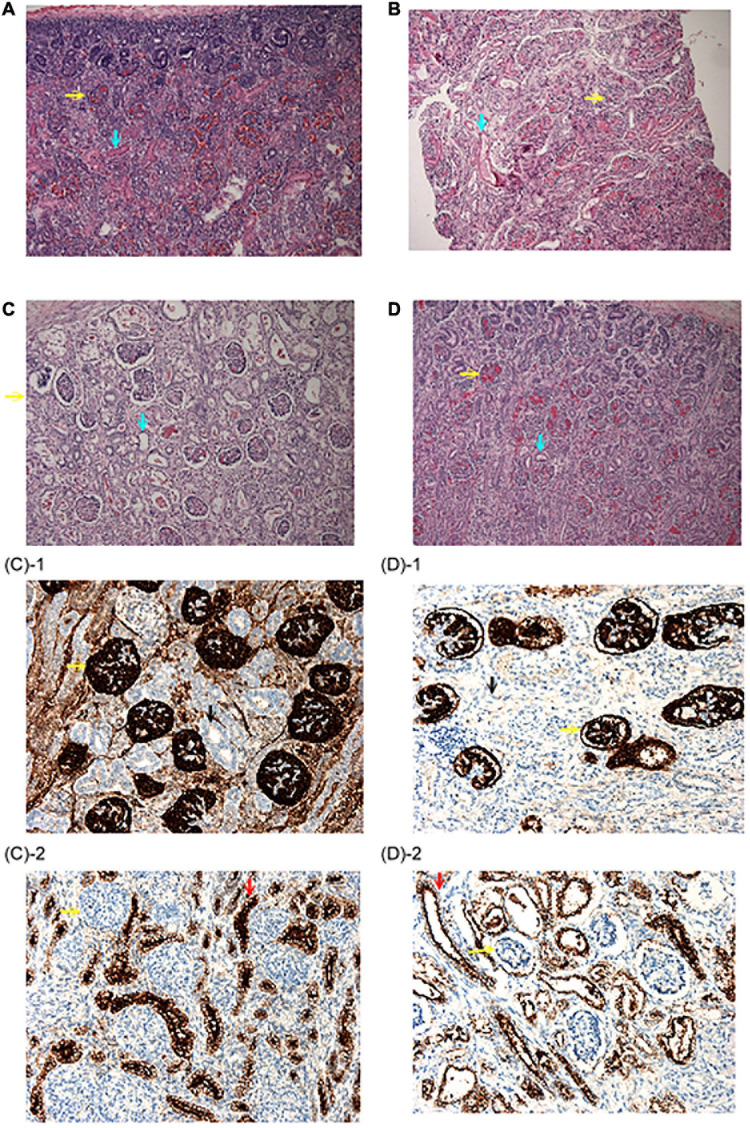
Renal histopathological findings. **(A)** Autopsy renal section from a fetus at 22 weeks due to congenital diaphragmatic hernia. Abundant proximal tubules were noted as the normal control. **(B)** Biopsy renal section from the patient 2–2 in family 2. Note that although proximal tubules could be seen, the cells were flat and had low cellularity. **(C,D)** Autopsy renal sections from the patient 1–1 and 1–2 in family 1. No well-developed proximal tubules could be identified. (H&E stain, original magnification: 100X) **(C-1,D-1)** were stained with anti-CD 10 antibody and no proximal tubules were illustrated. **(C-2,D-2)** Were stained with an anti-EMA antibody, which was positive for distal tubules. (Original magnification: 200X) Yellow arrow: Glomeruli; Blue arrow: proximal tubules; Black arrow: anti-CD 10(–) tubules; Red arrow: anti-EMA (+) distal tubules.

The 35-year-old woman, gravida 3 para 3, is a carrier of α-thalassemia, while her husband is a carrier of β-thalassemia (Figure 1A). There was no known family history of other genetic diseases. Her first pregnancy was uneventful and resulted in the cesarean delivery of a normal-term baby, who is currently a healthy 5-year-old child. Her second pregnancy (patient 1–1) was uncomplicated until 30 weeks of gestation when oligohydramnios was noted. She was referred to our hospital, and fetal ultrasound showed anhydramnios, absence of a bladder, and hepatic calcifications. Maternal blood tests for Cytomegalovirus (CMV) infection screening showed no CMV IgM antibodies. At 32 weeks of gestation, she underwent cordocentesis for karyotyping and SNP array analysis, the results of which were normal (46, XX). At 33 weeks of gestation, she underwent cesarean section due to fetal distress and previous cesarean section. A premature female baby was delivered with a birth body weight of 1,839 g (small-for-date) and Apgar score of 4–6. The initial blood pressure in the neonatal intensive care unit was 26/18 mmHg, which was not a response to dopamine and compatible with hypotension. One day after birth, veno-arterial extracorporeal membrane oxygenation (VA-ECMO) was used for frequent desaturation and poor cardiopulmonary function. When the baby was 3 days old, peritoneal dialysis was started for oliguria. The baby expired on the 7th day after birth and received an autopsy, where renal biopsy showed no obvious acute tubular necrosis or renal dysplasia. However, well-developed proximal tubules were hardly found (Figure 2C). Eight months later, the mother began her third pregnancy via natural conception (patient 1–2). At 16 weeks of gestation, karyotyping and SNP array analysis of the amniotic fluid both revealed normal findings (46, XX). However, at the 21st week of gestation, oligohydramnios developed. Amnioinfusion with 500 mL warmed saline was performed three times, at 21, 23, and 24 weeks, but the infused amniotic fluid disappeared 1 week after amnioinfusion. Therefore, trio-WES was performed, and compound heterozygous mutations were identified in the AGT gene. After genetic counseling, the couple decided to terminate the pregnancy. At 26 weeks of gestation, an expired, immature, female baby was delivered with a birth body weight of 904 gm. Autopsy of the patient revealed no proximal tubules of the kidney (Figure 2D).

### Family 2

The first pregnancy (patient 2–1) of a 33-year-old female was complicated by oligohydramnios since the 18th week of gestation. The baby was delivered at the 26th week of gestation and expired within 24 h after birth due to lung hypoplasia, hypotension, and anuria (Figure 1B). The mother’s second pregnancy was uneventful and ended with a full-term, healthy, female baby. However, oligohydramnios was noted again in the 26th week of gestation in the mother’s third pregnancy (patient 2–2). Both karyotyping and SNP-based array analysis showed normal findings, and detailed ultrasound did not reveal any structural anomalies. Amnioinfusion with 500 mL warmed saline was performed twice, but the amniotic fluid disappeared 1 week after amnioinfusion. Therefore, the pregnancy was terminated in the 32nd week of gestation. The birth body weight was 1,588 g, and the initial BP was 36/15 mmHg and decreased gradually. Anuria was noted after birth, and peritoneal dialysis was started on the 4th day after birth. The baby was found to have progressive periventricular leukomalacia during a series of brain sonography follow-ups, and amplitude integrated electroencephalography (aEEG) showed a discontinuous background with abnormal amplitude at 2 weeks of age. Because of the poor outcomes, a withdrawal ventilator was selected after do-not-resuscitate orders were completed and signed by the family on the 18th day after birth. Biopsy of renal sections showed a flat appearance and low cellularity of the proximal tubules (Figures 2A,B).

## Diagnostic Assessment

### Methods

Informed consent was obtained from the two families after the nature of the study was fully explained, and the study was approved by the Ethics Review Committee of National Taiwan University Hospital, Taipei, Taiwan (201919957RIN). The fetal genomic DNA (patient 1–1, 1–2, and 2–1) was extracted from amniotic fluid by amniocentesis. The Affymetrix CytoScan 750K SNP array analysis (Affymetrix Inc., Santa Clara, CA, United States) was used to rule out subtle genomic dosage changes. The present study used the NextSeq 500 Mid Output v2 Kit (300 cycles) (Illumina, San Diego, CA, United States) with a high depth of coverage for 4,000 medical exome genes associated with clinically relevant phenotypes. Genomic DNA paired-end libraries were constructed based on the manufacturer’s instructions using the NimbleGen SeqCap EZ Choice Library (Roche). The captured DNA libraries were enriched and then sequenced using the Illumina NextSeq 500 platform with an overall coverage depth > 10×. The clean reads from the Illumina NextSeq 500 were aligned to the human reference genome (GRCh37 + decoy). BAM and VCF files were generated by Genome Analysis Tool Kit (GATK V3.5, Broad Institute) (McKenna et al., 2010). Variants were first annotated by Variant Studio (V3.0, Illumina) and ANNOVAR (Wang et al., 2010). The pathogenicity of variants was checked by ClinVar^1^ and classified according to ACMG Guidelines (Richards et al., 2015). Germline copy number variation (CNV) was evaluated by GATK GermlineCNVCaller (Broad institute of Massachusetts, Cambridge, MA, United States). Reverse transcription-PCR (RT-PCR) of renal tissue for *AGT* mRNA expression was performed using primers designed in-house (Supplementary Figure 1 and Supplementary Table 2).

### Case Analysis

#### Family 1

**FIGURE 3:**
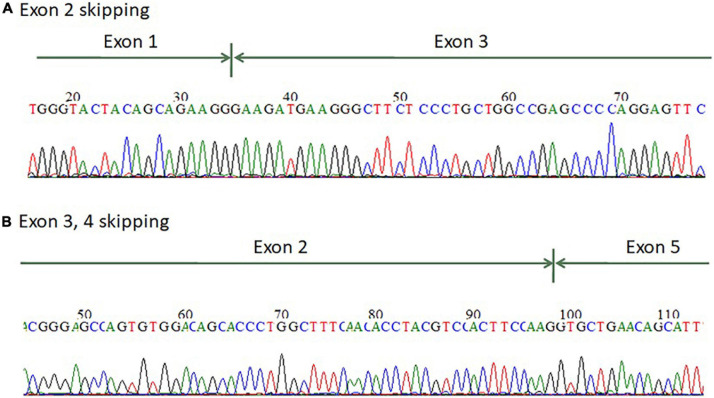
mRNA splicing analysis of the *AGT* gene from renal tissue of the terminated fetus showed that the fetus has **(A)** exon 2 skipping and **(B)** exon 3–4 skipping.

**FIGURE 4:**
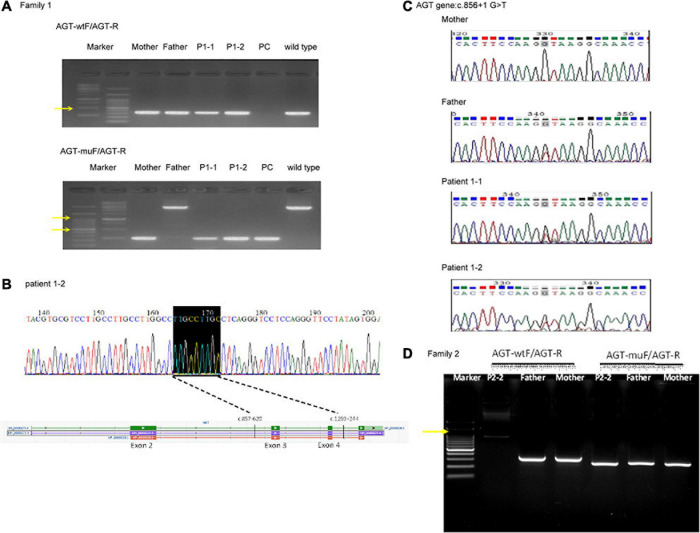
Genomic DNA analysis of the reported deletion in the Taiwanese population using primers as previously reported (Ma et al., 2019). **(A)** Genomic DNA analysis of family 1. *AGT*-wtF/*AGT*-R produced wild-type products (343 bp). *AGT*-muF/*AGT*-R yielded a 250 bp product for c.857-619_1269 + 243delinsTTGCCTTGC and an ∼3,000 bp product for the wild type. The result indicates that the father has a wild-type allele and the proband and mother are heterozygous for this mutation. Yellow arrows indicate 1,000 and 500 bp size markers. **(B)** Sequencing of the *AGT*-muF/*AGT*-R mutant product proved the c.857-619_1269 + 243delinsTTGCCTTGC change in patient 1–2. **(C)** Sanger sequencing result of gDNA PCR for family 1 at AGT gene c.856 + 1 G > T. **(D)** Genomic DNA analysis of family 2. PC, positive control.

For patients 1–2, we first searched for reported variants using our in-house software with genes that may involve in renal agenesis/oligohydramnios. A reported AGT pathogenic variant (c.856 + 1G > T) in Clinvar (Variation ID: 429788) was filtered in that was found from father (Figures 1C, 4C). Because AGT may explain the patient’s phenotype, we searched for another variant of AGT and found the patient had exon 3 and exon 4 deletion from mother and then solved this case (Figure 1C) within 1 week. In addition, searching for other candidate genes using HPO (Human Phenotype Ontology) terms did not found other candidate variants. To search for aberrant splicing, we performed in-house-designed 5-set RT-PCR for RNA from the proband’s kidney after termination and revealed two different transcriptions involving exon 2 skipping and exon 3–4 skipping (Figure 3 and Supplementary Figure 1). Further gDNA analysis of another reported deletion (Ma et al., 2019) confirmed the c.857-619_1269+ 243delinsTTGCCTTGC mutation in the first patient (Figures 4A,B and Supplementary Table 2).

#### Family 2

For patients 2–2 based on the first case’s experience, we focused on the AGT gene first and found the patient had exon 3 and exon 4 homozygous deletion from IGV. Also, searching for other candidate genes using HPO (Human Phenotype Ontology) terms did not found other candidate variants (Figure 1D). Further gDNA analysis confirmed the c.857-619_1269 + 243delinsTTGCCTTGC mutation in patient 2–2, and the parents were determined to be heterozygous carriers (Figure 4D).

## Discussion

Autosomal recessive renal tubular dysgenesis (ARRTD; OMIM#267430) is a severe fetal disorder characterized by the absence or poor development of proximal tubules, early-onset, and persistent anuria (leading to oligohydramnios and the Potter sequence), and ossification defects of the skull (Gubler, 2014). In most cases, neonatal death occurs due to pulmonary hypoplasia, anuria, and refractory arterial hypotension. Renal tubular dysgenesis (RTD) may result from genetic mutations in the gene encoding the major components of the renin-angiotensin-aldosterone system (RAAS), including angiotensinogen *(AGT*; MIM# 106150), renin (*REN*; MIM# 179820), angiotensin-converting enzyme, and angiotensin II receptor type 1 (*AGTR1*; MIM# 106165) (Gribouval et al., 2005, 2012). The diagnosis of ARRTD by traditional genetic sequencing, such as Sanger sequencing, is challenging and time-consuming. In family 2, the proband displayed a homozygous c.857-619_1269 + 243delinsTTGCCTTGC mutation in the *AGT* gene, and the parents were found to be heterozygous carriers by gDNA analysis. This mutation causes a subsequent premature stop codon in exon 5 (NP_000020.1:p.Gly286Valfs^∗^6), leading to a product shorter by 292 amino acids that contain 285 intact N-terminal amino acids of AGT. In family 1, the heterozygous deletion, including part of intron 2 (620 bp), exon 3, and exon 4, which are difficult or impossible to detect by the traditional PCR of each exon; therefore, rapid trio exome sequencing was helpful in the diagnosis of RTD with compound heterozygous mutations.

The prenatal diagnosis of ARRTD is quite complicated. Although histologic findings may facilitate diagnosis, fetal renal biopsy is challenging. Moldavsky (2009) reported that all 12 cases of RTD diagnosis were established postmortem. Three of the cases were diagnosed by autopsy material, while in the nine remaining cases, the diagnosis was made retroactively (in one case, 18 years after autopsy). Even though the two affected children, in this case, were sent for autopsy, pathologic diagnosis was not made until molecular diagnosis was confirmed.

The identified c.856 + 1G > T mutation in the *AGT* gene was localized in an evolutionarily conserved nucleotide (IVS856 + 1) of intron 2. Chen et al. (2019) reported a case of inheritance of the heterozygous c.856 + 1G > T mutation of the *AGT* gene in a 29-year-old male Chinese patient with multiple renal cysts and hypertension. This splice variant destroys the established splice donor site, and the ClinVar database predicted that this mutation might lead to an abnormal message or an abnormal protein product. The Web-based software MutationTaster predicted that this mutation was disease-causing and capable of causing splice site changes and affecting protein features. The c.856 + 1G > T variant is not observed at a significant frequency in large population cohorts (Genomes Project Consortium, Auton et al., 2015; Lek et al., 2016). In addition, the c.857-619_1269 + 243delinsTTGCCTTGC mutation identified in the *AGT* gene was previously reported in seven Taiwanese families (Ma et al., 2019; Min-Hua et al., 2020). This suggests that this allele may be the result of a founder effect. The deletion results in partial loss of the serpin domain of the mature AGT protein (Wu et al., 2011).

We ran a omozygosity plot on patient 2 using AutoMap (Quinodoz et al., 2021). The Runs of Homozygosity (ROH) region in patient 2 was 43.13 Mb in total (Supplementary Figure 2). According to other literature (McQuillan et al., 2008), outbred individuals never carry ROH over 4 Mb in length. Since there is a possibility that parents may be distantly related, we calculated the Inbreeding Coefficient (F_*ROH*_) by the percentage of the genome that is homozygous (ROH length > 1Mb) compared with the total autosomal genomic (approximately 2,691 Mb for GRCh37/hg19) (Matalonga et al., 2020). In patient 2, the F_*ROH*_ is 1.60% (43.13 Mb/2691 Mb) was classified as uncertain for consanguinity (0.05 < consanguinity probability < 0.5) according to the model (Matalonga et al., 2020). On the other hand, the inbreeding coefficient = 1.60% implied the parents may have a relation five generations ago (∼120 years).

The prognosis of oligohydramnios is variable, and an accurate and prompt diagnosis could be of great importance in prenatal decision-making or postnatal management. Because of the diversity of etiologies in oligohydramnios, trio-WES may provide an efficient and timely molecular diagnosis prenatally. The genetic results were obtained within 1 week by tri-WES analysis (Wu et al., 2019). Several studies have discussed the advantage of trio-WES with respect to the efficiency of variation screening (Al-Mubarak et al., 2017; Hegde et al., 2017; Fu et al., 2018). Best S et al. reviewed 31 prenatal WES studies and the diagnostic rates ranged from 6.2 to 80% across the 16 studies with five or more fetuses tested (Best et al., 2018). Prenatal WES studies also have the potential to improve our understanding of lethal genetic disorders presenting with fetal abnormalities, where the full phenotype is often unknown. NGS approaches will facilitate gene discovery and may reveal Mendelian inheritance were previously missed. A family with recurrent intrauterine growth restriction and multiple abnormalities was identified as having a new lethal ciliary disorder by trio-WES when autosomal recessive truncating mutations in the *KIF14* gene that segregated with the phenotype were detected (Filges et al., 2014). According to our reported case, the diagnosis of ARRTD by trio-WES may confirm the poor prognosis of the fetus and avoid further amnioinfusion or treatment to no avail. Diagnosing a lethal genetic condition before birth gives parents time to make choices about pregnancy management, including termination within local legal limits, or discuss palliative care before or after delivery. A prenatal diagnosis may therefore reduce extended neonatal hospital stays while awaiting a diagnosis. Furthermore, a genetic diagnosis also allows counseling about recurrence risks and can facilitate preparation for prenatal testing or preimplantation genetic diagnosis in subsequent pregnancies.

## Conclusion

In conclusion, we used trio-WES to identify mutations segregating in the *AGT* gene in two non-consanguineous Taiwanese families presenting with recurrent prenatal oligohydramnios. Prompt prenatal diagnosis may facilitate pregnancy decision-making, and a defined single gene etiology facilitates genetic counseling related to recurrence risk, opening avenues for preimplantation genetic diagnosis and future prenatal testing.

## Data Availability Statement

The datasets presented in this study can be found in online repositories. The names of the repository/repositories and accession number(s) can be found in the article/Supplementary Material.

## Ethics Statement

The studies involving human participants were reviewed and approved by the Ethics Review Committee of National Taiwan University Hospital, Taipei, Taiwan (201919957RIN). The patients/participants provided their written informed consent to participate in this study. Written informed consent was obtained from the individual(s) for the publication of any potentially identifiable images or data included in this article.

## Author Contributions

S-YL contributed to drafting the manuscript and revision. G-TC, C-HH, C-NL, and T-AY cared for the patients and collected clinical data. W-CL and Y-MJ helped with the H&E staining and pathologic analysis. KC, Y-HC, W-LH, and N-CL analyzed and interpreted patient data. I-JT and N-CL contributed to data collection and the revision of the manuscript. All authors read and approved the final manuscript.

## Conflict of Interest

The authors declare that the research was conducted in the absence of any commercial or financial relationships that could be construed as a potential conflict of interest.

## Footnotes

1 https://www.ncbi.nlm.nih.gov/clinvar/

## Supplementary Material

Supplementary Figure 1 Primer designs for mRNA expression analysis of the AGT gene.

Supplementary Figure 2 Homozygous Region for patient 2 analyzed by AutoMap v1.0 with threshold > 1.0Mb. A total 43.12Mb of autosomes are in homozygous regions (blue bars) with median 1.44Mb (range 1.04−4.08 Mb). The AGT gene locates on chr1:230,838,269−230,850,336 that is not in the reported homozygous region.

Supplementary Table 1 The differential diagnosis of oligohydramnios (HP:0001562) from Human Phenotype Ontology.

Supplementary Table 2 Primer sequences for mRNA analysis (Figure 2).
