# Supplementary figures and images for: Rapid Trio Exome Sequencing for Autosomal Recessive Renal Tubular Dysgenesis in Recurrent Oligohydramnios
Source: Front Genet. 2021 Jun 21;12:606970. doi: 10.3389/fgene.2021.606970 (PMC8255961; doi:10.3389/fgene.2021.606970)

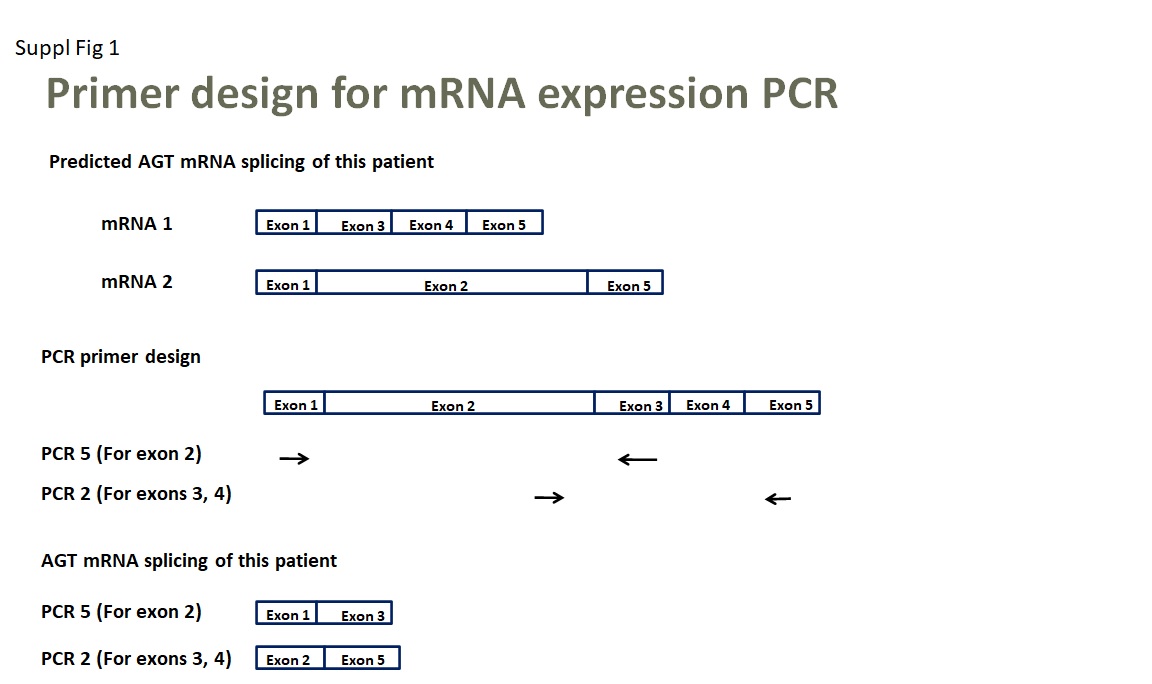

Supplement: Supplementary Figure 1 — Primer designs for mRNA expression analysis of the AGT gene. [file Image_1.JPEG]

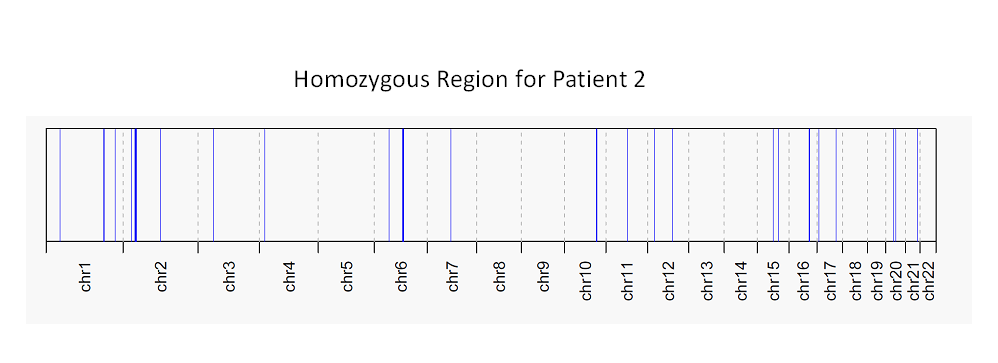

Supplement: Supplementary Figure 2 — Homozygous Region for patient 2 analyzed by AutoMap v1.0 with threshold > 1.0Mb. A total 43.12Mb of autosomes are in homozygous regions (blue bars) with median 1.44Mb (range 1.04−4.08 Mb). The AGT gene locates on chr1:230,838,269−230,850,336 that is not in the reported homozygous region. [file Image_2.TIF]
