# Supplementary material for: Rapid Trio Exome Sequencing for Autosomal Recessive Renal Tubular Dysgenesis in Recurrent Oligohydramnios
Source: Front Genet. 2021 Jun 21;12:606970. doi: 10.3389/fgene.2021.606970 (PMC8255961; doi:10.3389/fgene.2021.606970)
Supplement: Supplementary Table 1 — The differential diagnosis of oligohydramnios (HP:0001562) from Human Phenotype Ontology. [file Table_1.docx]

**Supplementary Table 1. The differential diagnosis of oligohydramnios (HP:0001562) from Human Phenotype Ontology.**

| GENE_SYMBOL | DISEASE_IDS |
| --- | --- |
| ACE | OMIM:267430 |
| AGT | OMIM:145500,OMIM:267430 |
| AGTR1 | OMIM:145500,OMIM:267430 |
| ALB | ORPHA:86816,OMIM:616000 |
| ALG8 | OMIM:617874,ORPHA:79325,OMIM:608104 |
| ALG9 | OMIM:608776,OMIM:263210,ORPHA:79328 |
| ALX4 | OMIM:613451,ORPHA:60015,ORPHA:35093,OMIM:609597,ORPHA:52022,ORPHA:228390,OMIM:615529 |
| AMER1 | OMIM:300373,ORPHA:2780 |
| ASCL1 | ORPHA:99803,OMIM:209880 |
| ATRX | ORPHA:100075,ORPHA:231401,OMIM:300448,ORPHA:93973,ORPHA:93974,OMIM:301040,ORPHA:93971,ORPHA:847,  ORPHA:93970,OMIM:309580,ORPHA:93972 |
| B3GALT6 | OMIM:615349,OMIM:271640,ORPHA:536467,ORPHA:93359 |
| B9D1 | OMIM:614209,ORPHA:475,OMIM:617120,ORPHA:564 |
| B9D2 | OMIM:614175,ORPHA:564 |
| BMPER | OMIM:608022,ORPHA:66637 |
| BNC2 | OMIM:618612,ORPHA:93110 |
| BRCA1 | OMIM:114480,ORPHA:84,OMIM:617883,OMIM:604370,ORPHA:145,ORPHA:168829,ORPHA:1333 |
| BRCA2 | OMIM:114480,OMIM:176807,ORPHA:84,OMIM:605724,OMIM:613029,ORPHA:145,OMIM:612555,OMIM:155255,ORPHA:654,  OMIM:194070,OMIM:613347,ORPHA:1333 |
| BRIP1 | OMIM:114480,ORPHA:84,OMIM:609054,ORPHA:145 |
| BUB1B | ORPHA:1052,OMIM:176430,OMIM:114500,OMIM:257300 |
| C1QBP | OMIM:617713 |
| CC2D2A | ORPHA:1454,ORPHA:2318,OMIM:612284,OMIM:612285,ORPHA:564 |
| CDKN1C | ORPHA:397590,ORPHA:85173,OMIM:130650,ORPHA:436144,OMIM:614732 |
| CEP290 | ORPHA:3156,ORPHA:65,OMIM:610188,OMIM:615991,ORPHA:110,OMIM:611755,ORPHA:2318,OMIM:610189,OMIM:611134,ORPHA:564 |
| CEP55 | OMIM:236500,ORPHA:564 |
| CERT1 | OMIM:616351 |
| CHRM3 | OMIM:100100,ORPHA:2970 |
| COG5 | ORPHA:263487,OMIM:613612 |
| COQ2 | OMIM:607426,ORPHA:255249,OMIM:146500,ORPHA:227510,ORPHA:98933 |
| COQ7 | OMIM:616733 |
| COX14 | OMIM:619053 |
| CPT2 | OMIM:614212,ORPHA:228305,OMIM:600649,ORPHA:228308,ORPHA:228302,OMIM:608836,OMIM:255110 |
| CSPP1 | ORPHA:397715,OMIM:615636,ORPHA:475,ORPHA:564 |
| DALRD3 | OMIM:618910,ORPHA:442835 |
| DDX6 | ORPHA:528084,OMIM:618653 |
| DHPS | OMIM:618480 |
| DOCK6 | OMIM:614219,ORPHA:974 |
| DONSON | OMIM:617604,OMIM:251230 |
| DYRK1A | ORPHA:268261,ORPHA:464311,OMIM:614104 |
| DZIP1L | OMIM:617610,ORPHA:731 |
| EBF3 | OMIM:617330 |
| EFEMP2 | OMIM:614437,ORPHA:90349 |
| ERCC4 | ORPHA:910,ORPHA:84,ORPHA:220295,ORPHA:90321,OMIM:610965,OMIM:278760,OMIM:615272 |
| ERGIC1 | OMIM:208100,ORPHA:1143 |
| EXOSC9 | OMIM:618065 |
| FANCA | ORPHA:84,OMIM:227650 |
| FANCB | ORPHA:84,ORPHA:3412,OMIM:300514,OMIM:314390 |
| FANCC | ORPHA:84,OMIM:227645 |
| FANCD2 | ORPHA:84,OMIM:227646 |
| FANCE | OMIM:600901,ORPHA:84 |
| FANCF | OMIM:603467,ORPHA:84 |
| FANCG | OMIM:614082,ORPHA:84 |
| FANCI | ORPHA:84,OMIM:609053 |
| FANCL | ORPHA:84,OMIM:614083 |
| FANCM | ORPHA:84,ORPHA:399805,OMIM:618086,OMIM:618096 |
| FARSA | OMIM:619013 |
| FARSB | OMIM:613658 |
| FBLN5 | OMIM:614434,ORPHA:90348,OMIM:219100,OMIM:608895,ORPHA:90349 |
| FBN1 | OMIM:616914,ORPHA:2462,ORPHA:2084,ORPHA:969,OMIM:102370,OMIM:184900,OMIM:608328,OMIM:604308,ORPHA:284979,  OMIM:154700,OMIM:129600,ORPHA:3449,OMIM:614185,ORPHA:91387,ORPHA:2833,ORPHA:1885 |
| FGF20 | OMIM:615721,ORPHA:1848 |
| FXR1 | OMIM:618823,OMIM:618822 |
| GATA6 | OMIM:614475,OMIM:614474,OMIM:217095,ORPHA:3303,ORPHA:1330,OMIM:187500,ORPHA:2255,OMIM:600001,  ORPHA:2140,ORPHA:99103 |
| GLI3 | OMIM:174700,OMIM:146510,ORPHA:380,ORPHA:672,OMIM:174200,OMIM:175700,ORPHA:93322,ORPHA:36 |
| GMPPB | OMIM:615352,ORPHA:353327,OMIM:615350,ORPHA:370959,ORPHA:588,ORPHA:370968,ORPHA:363623,OMIM:615351 |
| GNPTAB | OMIM:252500,OMIM:252600,ORPHA:576 |
| GREB1L | OMIM:617805,ORPHA:1848 |
| H19 | ORPHA:2128,ORPHA:231140,ORPHA:654,OMIM:194070,ORPHA:231144 |
| HBA1 | OMIM:140700,OMIM:613978,ORPHA:98791,ORPHA:163596,OMIM:604131 |
| HBA2 | OMIM:140700,OMIM:613978,ORPHA:98791,ORPHA:163596,OMIM:604131 |
| HMGA2 | ORPHA:397590,ORPHA:94063,ORPHA:99971,OMIM:618908 |
| HNF1B | ORPHA:261265,OMIM:125853,ORPHA:93111,OMIM:144700,OMIM:137920 |
| HSPA9 | OMIM:616854,OMIM:182170 |
| HYMAI | ORPHA:96191,ORPHA:99886,OMIM:601410 |
| IARS1 | OMIM:617093,ORPHA:541423 |
| IGF2 | ORPHA:397590,OMIM:180860,ORPHA:2128,OMIM:130650,ORPHA:231140,OMIM:616489,OMIM:194070,ORPHA:231144 |
| INVS | ORPHA:3156,OMIM:602088 |
| ITGA8 | OMIM:191830,ORPHA:1848 |
| KIF14 | OMIM:616258,OMIM:617914,ORPHA:2512 |
| LARS2 | OMIM:617021,OMIM:615300 |
| LIFR | ORPHA:3206,OMIM:601559 |
| MAD2L2 | ORPHA:84,OMIM:617243 |
| MBTPS2 | OMIM:308205,ORPHA:659,OMIM:301014,OMIM:308800,ORPHA:2273,ORPHA:85284,OMIM:300918 |
| MKS1 | ORPHA:110,ORPHA:475,OMIM:615990,ORPHA:220493,OMIM:617121,ORPHA:564,OMIM:249000 |
| MYH3 | OMIM:193700,OMIM:618436,OMIM:178110,OMIM:618469,ORPHA:1146,ORPHA:2990,ORPHA:1147,ORPHA:2053 |
| NALCN | OMIM:616266,ORPHA:1146,ORPHA:1147,ORPHA:371364,ORPHA:2053,OMIM:615419 |
| NEK8 | OMIM:613824,OMIM:615415 |
| NEK9 | OMIM:617022,OMIM:614262,ORPHA:64754,OMIM:617025 |
| NPHP3 | ORPHA:3156,OMIM:267010,OMIM:604387,OMIM:208540,ORPHA:3032 |
| OSGEP | ORPHA:2065,OMIM:617729 |
| PALB2 | OMIM:114480,ORPHA:84,OMIM:610832,ORPHA:145,ORPHA:1333 |
| PBX1 | OMIM:617641 |
| PDSS2 | OMIM:614652,ORPHA:255249 |
| PGAP2 | ORPHA:247262,OMIM:614207 |
| PGAP3 | OMIM:615716,ORPHA:247262 |
| PHOX2B | ORPHA:99803,ORPHA:2151,ORPHA:635,ORPHA:661,OMIM:209880,OMIM:613013 |
| PIGL | ORPHA:3474,ORPHA:247262,OMIM:280000 |
| PIGO | ORPHA:247262,OMIM:614749 |
| PIGV | ORPHA:247262,OMIM:239300 |
| PIGW | ORPHA:247262,OMIM:616025 |
| PIGY | ORPHA:247262,OMIM:616809 |
| PKHD1 | ORPHA:731,OMIM:263200,ORPHA:53035 |
| PLAG1 | ORPHA:397590,OMIM:618907,OMIM:181030 |
| POR | OMIM:613571,OMIM:207410,OMIM:201750,ORPHA:95699 |
| PUF60 | OMIM:615583,ORPHA:508498,ORPHA:508488 |
| RAD51 | OMIM:114480,ORPHA:84,OMIM:614508,ORPHA:145,ORPHA:238722,OMIM:617244 |
| RAD51C | OMIM:613399,ORPHA:84,ORPHA:145,OMIM:613390 |
| REN | OMIM:613092,OMIM:267430 |
| RET | OMIM:142623,ORPHA:99803,OMIM:162300,OMIM:155240,ORPHA:29072,OMIM:209880,OMIM:171300,OMIM:171400,ORPHA:276621,  ORPHA:1848,ORPHA:388 |
| RFWD3 | OMIM:617784,ORPHA:84 |
| RNU4ATAC | ORPHA:1824,OMIM:226960,ORPHA:353298,ORPHA:2636,OMIM:210710,OMIM:616651 |
| RPGRIP1 | ORPHA:65,OMIM:613826,OMIM:608194,ORPHA:1872,ORPHA:564 |
| RPGRIP1L | OMIM:611560,ORPHA:1454,ORPHA:220497,OMIM:611561,ORPHA:564 |
| SEC24D | OMIM:616294,ORPHA:2050 |
| SETD1A | OMIM:618832,OMIM:619056 |
| SLC25A24 | OMIM:612289,ORPHA:2963,ORPHA:2095 |
| SLX4 | ORPHA:84,OMIM:613951 |
| SNRPN | OMIM:176270,ORPHA:411515,ORPHA:177910,ORPHA:177904,ORPHA:177901,ORPHA:177907,ORPHA:98754,OMIM:209850 |
| TALDO1 | OMIM:606003,ORPHA:101028 |
| TBCK | ORPHA:488632,OMIM:616900 |
| TCTN2 | ORPHA:475,OMIM:616654,OMIM:613885,ORPHA:564 |
| TCTN3 | OMIM:614815,OMIM:258860,ORPHA:2754,ORPHA:2753 |
| TMEM107 | OMIM:617562,OMIM:617563,ORPHA:564 |
| TMEM216 | OMIM:603194,ORPHA:2754,ORPHA:2318,OMIM:608091,ORPHA:564 |
| TMEM231 | OMIM:614970,ORPHA:2752,OMIM:615397,ORPHA:2318,ORPHA:564 |
| TMEM67 | ORPHA:1454,OMIM:615991,OMIM:607361,ORPHA:475,OMIM:613550,OMIM:602152,OMIM:216360,OMIM:610688,ORPHA:564 |
| TMEM70 | OMIM:614052,ORPHA:1194 |
| TRIP4 | OMIM:616866,OMIM:617066,ORPHA:486815 |
| UBE2A | ORPHA:163956,OMIM:300860 |
| UBE2T | ORPHA:84,OMIM:616435 |
| WDPCP | OMIM:615992,ORPHA:110,OMIM:217085,ORPHA:564,ORPHA:1338 |
| WDR73 | OMIM:251300,ORPHA:2065,ORPHA:83472 |
| WNT4 | OMIM:611812,ORPHA:139466,OMIM:158330,ORPHA:247768 |
| XRCC2 | ORPHA:84,ORPHA:399805,OMIM:619145,OMIM:617247,OMIM:619146 |
