# Supplementary material for: Rapid Trio Exome Sequencing for Autosomal Recessive Renal Tubular Dysgenesis in Recurrent Oligohydramnios
Source: Front Genet. 2021 Jun 21;12:606970. doi: 10.3389/fgene.2021.606970 (PMC8255961; doi:10.3389/fgene.2021.606970)
Supplement: Supplementary Table 2 — Primer sequences for mRNA analysis (Figure 2). [file Table_2.docx]

**Supplementary Table 2** Primer sequences for mRNA analysis (Figure 2)

| **Name** | **Forward** | **Reverse** |
| --- | --- | --- |
| **PCR2 (For exons 3,4)** | 5’-gaactggatgttgctgctga-3’ | 5’-atccgcttcaagctcaaaaa-3’ |
| **PCR5 (For exon 2)** | 5-tggatgaaaaggccctacag-3’ | 5’-tcccttggaagtggacgtag-3’ |
